# Development of the multi-attribute Adolescent Health Utility Measure (AHUM)

**DOI:** 10.1186/1477-7525-10-102

**Published:** 2012-08-28

**Authors:** Kathleen M Beusterien, Jean-Ezra Yeung, Francis Pang, John Brazier

**Affiliations:** 1 Oxford Outcomes Inc, an ICON plc Company, 7315 Wisconsin Ave. Ste 250 W, Bethesda, MD, 20814, UK; 2 Shire AG (Shire), Route de Crassier, 7, 1262, Eysins, Switzerland; 3 Health Economics and Decision Science, School of Health and Related Research, University of Sheffield, Regent Court, 30 Regent Street, Sheffield, S1 4DA, UK

## Abstract

**Objective:**

Obtain utilities (preferences) for a generalizable set of health states experienced by older children and adolescents who receive therapy for chronic health conditions.

**Methods:**

A health state classification system, the Adolescent Health Utility Measure (AHUM), was developed based on generic health status measures and input from children with Hunter syndrome and their caregivers. The AHUM contains six dimensions with 4–7 severity levels: self-care, pain, mobility, strenuous activities, self-image, and health perceptions. Using the time trade off (TTO) approach, a UK population sample provided utilities for 62 of 16,800 AHUM states. A mixed effects model was used to estimate utilities for the AHUM states. The AHUM was applied to trial NCT00069641 of idursulfase for Hunter syndrome and its extension (NCT00630747).

**Results:**

Observations (i.e., utilities) totaled 3,744 (12*312 participants), with between 43 to 60 for each health state except for the best and worst states which had 312 observations. The mean utilities for the best and worst AHUM states were 0.99 and 0.41, respectively. The random effects model was statistically significant (p < 0.0001; adjusted R2 = 0.361; RMSE = 0.194). When AHUM utilities were applied to the idursulfase trial, mean utilities in the idursulfase weekly and placebo groups improved +0.087 and +0.006, respectively, from baseline to week 53. In the extension, when all patients received idursulfase, the utilities in the treatment group remained stable and the placebo group improved +0.039.

**Discussion:**

The AHUM health state classification system may be used in future research to enable calculation of quality-adjust life expectancy for applicable health conditions.

## Introduction

An important innovation in cost effectiveness analysis in health care has been the development of the quality adjusted life year (QALY) that combines longevity with quality of life. It achieves this by assigning a preference value (utility weight) to each health state experienced by a patient on a scale where 0.0 reflects being dead and 1.0 reflects full health [1]. Evaluation of the incremental cost per QALY gained, or cost-utility analyses, is now a standard type of cost-effectiveness analysis [2,3].

The last decade has seen increasing use made of generic utility measures that are assigned preference weights based on the general population perspective, as recommended by the US Panel on Cost-Effectiveness in Health and Medicine and used by the National Institute for Health and Clinical Excellence (NICE) [4,5]. Examples of such measures include the EQ-5D and the SF-6D [6,7]. These are multi-attribute scales based on a generic health state classification where health is described across multi-level dimensions of health applicable across different health conditions. These measures come with a set of preference weights obtained from members of the general public using a preference elicitation technique like the time trade off (TTO) approach. All such multi-attribute measures, however, have descriptive systems that are derived from adult populations with the exception of the Heath Utility Index Mark 2, and thus additional attributes that are particularly relevant to child health, including autonomy and body image, may not be captured [2].

Based on a critical review of published cost-utility studies in child health, among the approaches cited for implementing cost-utility analysis in pediatric populations include the explicit development of a generic instrument designed to be applicable across child and adult populations [2]. The objective of the current study was to develop a multi-attribute measure, the adolescent health utility measure (AHUM), that focuses on key impacts of treatment for chronic conditions among older children and adolescents. Researchers would then be able to use this measure, either prospectively collecting AHUM data or mapping the AHUM utilities to patient-reported outcomes (PRO) measure data that already have been collected, to quality-adjust life expectancy in future economic evaluations that involve interventions for chronic conditions that may be experienced in childhood.

## Methods

Development of the AHUM involved three steps. The first step was the selection of the health attributes and respective levels, which, in different combinations, characterized the AHUM health states. The second step was to obtain values (utility weights) for a sample of AHUM health states from the general adult population, as preferred by NICE. The third step was to develop a statistical model to estimate mean values for all of the AHUM health states. Finally, we applied the AHUM to a clinical trial sample of children with Hunter syndrome and compared AHUM utility scores over time between the treatment (idursulfase) and placebo groups.

### Selection of AHUM attributes

In selecting the attributes (dimensions) for the AHUM, we considered the concepts captured by common generic measures such as the EQ-5D (mobility, self-care, usual activities, pain/discomfort and anxiety/depression) and the SF-6D (physical functioning, role limitations, social functioning, bodily pain, mental health, and tiredness/vitality). In addition, a review of the literature focusing on impacts of childhood conditions identified key respective concerns. Specifically, Norrby and colleagues [8] used the Child Health Questionnaire (CHQ) to evaluate health-related quality of life among children with asthma, diabetes, juvenile chronic arthritis, or short stature. The CHQ scales showing detrimental effects that varied across conditions included physical functioning, bodily pain, self-esteem, and general health. Another study of adolescents with short stature showed that they may experience detriments in perceived competence at school, athletic ability, and trait anxiety [9].

In addition to this review, disease impacts identified by children (and caregivers) with Hunter syndrome informed the identification of the AHUM domains. Obtaining input from children with Hunter syndrome was useful because this condition has potentially wide-ranging impacts on health-related quality of life. Specifically, Hunter syndrome can cause coarse facial features, cardiovascular disorders, short stature, joint stiffness, skeletal deformities and mental retardation; eventually, patients become wheelchair-bound and ventilator dependent [10]. A total of 27 parents and 11 subjects with Hunter syndrome between 12 and 18 years of age were interviewed on the impacts of Hunter syndrome and treatment on daily life. Based on these interviews, key topics or themes were identified through qualitative analysis of the data. Specifically, the main areas of health identified by the children and caregivers were problems with: 1) lower body tasks such as walking (mobility); 2) upper body tasks such as eating, buttoning a shirt (self-care, e.g., dressing etc.); 3) sleeping; 4) schoolwork; 5) dissatisfaction with physical activities/sports; 6) breathing; and 7) satisfaction with current treatment/perception of making progress on treatment.

Based on these concepts and those identified in the literature, six dimensions with different respective severity levels were identified for the AHUM health state classification system: self-care, pain, limitations in walking around (mobility), perception of ability to do strenuous activities, self-image, and health perceptions (current health and health compared to one year ago (Table 1). These concepts were those identified in the qualitative research, with the exception of sleeping, schoolwork, breathing, and trait anxiety, which were determined to be too narrowly focused for inclusion in a health state classification system capturing overall health-related quality of life. The selected AHUM concepts were augmented with self-image and pain, two concepts that were identified in the literature review, and the latter of which appears in both the SF-6D and EQ-5D. The AHUM domains and levels utilize similar concepts, adapted to the present tense, as those used in existing general health status questionnaires, such as the 20-item Medical Outcomes Study (MOS) questionnaire (RAND), 36-item Short-Form Health Survey, CHQ, and the more specific Rosenberg Self-Esteem scale [11-14]). 

**Table 1:** AHUM Health State Classification System

**Level**	**Descriptions**
	**Self-care**
1	You are not limited in eating, dressing, bathing, or using the toilet
2	You are limited a little in eating, dressing, bathing, or using the toilet
3	You are limited moderately in eating, dressing, bathing, or using the toilet
4	You are limited a lot in eating, dressing, bathing, or using the toilet
	**Pain**
1	You have no pain
2	You have very mild pain
3	You have mild pain.
4	You have moderate pain
5	You have severe pain
6	You have very severe pain
	**Limitations walking around (mobility)**
1	You are not limited in walking around or going to school/work
2	You are limited a little in walking around or going to school/work
3	You are limited moderately in walking around or going to school/work
4	You are limited a lot in walking around or going to school/work
	**Perceptions of strenuous activities**
1	You feel very good about your ability to do strenuous activities (like running or participating in sports).
2	You feel somewhat good about your ability to do strenuous activities (like running or participating in sports).
3	You feel neutral (neither good or bad) about your ability to do strenuous activities (like running or participating in sports).
4	You feel somewhat bad about your ability to do strenuous activities (like running or participating in sports).
5	You feel very bad about your ability to do strenuous activities (like running or participating in sports).
	**Self-image**
1	On the whole, you feel very good about your body.
2	On the whole, you feel somewhat good about your body.
3	On the whole, you feel neutral (neither good or bad) about your body.
4	On the whole, you feel somewhat bad about your body.
5	On the whole, you feel very bad about your body.
	**Health perceptions**
1	Your health is better now than one year ago, and you feel like you are in very good to excellent health.
2	Your health is better now than one year ago, and you feel like you are in good health.
3	Your health is the same now as one year ago, and you feel like you are in very good to excellent health.
4	Your health is the same now as one year ago, and you feel like you are in good health.
5	Your health is about the same or somewhat better now than one year ago, and you feel like you are in fair or poor health.
6	Your health is worse now than one year ago, and you feel like you are in good to excellent health.
7	Your health is worse now than one year ago, and you feel like you are in fair or poor health.

The omission of a dimension focusing on anxiety or emotional impact was intentional. During utility elicitation exercises like time trade off (TTO), respondents are asked to value being in different health states. We believe that, inherent in such evaluations is consideration of the emotional distress of being in the selected health state, and thus the respective emotional impact would be factored into the ultimate utility value for the selected health state. As such, adding a dimension on anxiety to the health state classification system would be redundant. We do not wish to dictate how much anxiety or distress a subject attaches to the different AHUM health states; rather, we anticipate that this emotional impact is reflected in the utility values.

### Derivation of preference weights

The different combinations of the attribute levels in Table 1 (e.g., 111111, 121133, etc.) reflect different possible health states; the classification system defines 16,800 health states. To estimate utility weights (preference values) for these health states, a cross-sectional survey was conducted in the UK general population using the time trade-off (TTO) approach. The TTO exercise is a widely accepted technique that yields preference weights on a scale from 0.0, reflecting being dead, to 1.0, reflecting full health [15]. The target sample was 300 people in the UK general population (England and Scotland). Six trained interviewers located in England and Scotland recruited study participants through word-of-mouth and the Oxford Outcomes’ general population panel. All study participants met the following eligibility criteria: resident of UK, 18 years of age or older, adequate written and oral fluency in English, and willing and able to provide written informed consent. All study participants were compensated £25 for their time. The study protocol was approved by Independent IRB (Plantation, FL, USA).

Given the vast number of possible health states that can be derived from the different combinations of levels across the AHUM domains, an orthogonal subset of 49 health states generated by Orthoplan in SPSS, as well as an additional 11 randomly selected states, were selected for inclusion in the TTO interviews. Each interview included obtaining utilities for 12 health states, including 10 of the 60 selected health states plus the best state (most favorable level of each domain: 111111) and the worst state (least favorable level of each domain: 464557). The best health state was included in the exercise to evaluate the magnitude of the difference in utility values between this state and full health. To increase precision in utility estimation for each state, the plan was to obtain values from at least 40 study participants for each health state. As discussed by Brazier et al. [16,17], the average values obtained from 10 respondents are likely to be very unreliable. With a target sample size of 300, the 6 study interviewers could administer sets of 10 unique health states to at approximately 33 study participants, yielding at least 40 TTO values for each health state. All health states were labeled with symbols rather than numbers to avoid imposing any kind of hierarchy with respect to their order.

During the TTO interviews, subjects were first asked to review 13 health states (10 assigned health states, the best and worse health state, and full health) and rank them from most favorable to least favorable to help familiarize the participant with the health states. Subsequently, for each health state (except for full health), the TTO exercise was conducted; the health states were shuffled, randomizing the order in which the health states were presented. Specifically, for each state, the respondent was asked to imagine living in the health state as described for 10 years or they can trade off some time to live in full health. The objective of the interview is to identify the maximum amount of the 10 years the respondent is willing to trade off to live in full health. The interviewer followed a standardized interview guide and used a prop to illustrate the comparison between the number of years in the selected health state and the number of years in full health. To minimize starting point bias, the time traded-off was alternated in 1 year increments from the most extreme to least extreme; once the number of years was identified, the interviewer further probed using months. In the TTO interviews, subjects sometimes found particular health states hard to imagine because of seemingly contradictory domain levels (e.g., how can you be very limited in walking around or going to school, yet feel that you are in good to excellent health?). In these scenarios, participants were asked to focus on those dimensions that mattered the most to them and try their best to imagine the scenario.

To calculate the health utility for each health state, the maximum number of years that the respondent was willing to trade off to live in full health was subtracted from 10 and this result was divided by 10 to arrive at the utility of the health state, where a score of 0.0 score reflects being dead, and a 1.0 score reflects full health. It should be noted that utilities may be worse than being dead (i.e., utilities can have negative values); however, the TTO approach used in this study did not allow for health states to be rated as worse than being dead as it was thought that this would occur only rarely; thus, no negative utilities were obtained. If a respondent considered a health state as worse than being dead, the health state was assigned a utility of 0.

### Modeling health state values

The aim of developing a model was to enable the estimation of all possible health states (all the possible combinations of levels) that can be derived from the AHUM health state classification system, which yields 16,800 unique health states. A mixed effects model was performed that included fixed effects for the levels of each AHUM domain and random effects for each subject. The model also included a fixed effect interaction term for being at the worse level of any domain, which is a common method to allow for non-linearities in modeling preference data [6,7,16,17]. Thus, the model included 26 fixed effects in total. No variables other than the AHUM levels and the subject were included in the model. Analyses were performed using SAS v9.2.

### Application of AHUM utilities to pediatric clinical trial

The model for estimation of AHUM utilities was applied to a phase II/III, randomized, double-blind, placebo-controlled 53-week study (NCT00069641) evaluating the safety and efficacy of weekly and every other week dosing regimens of iduronate-2-sulfatase enzyme replacement therapy in patients with Hunter syndrome [18] and its extension study (NCT00630747) [19]. In conjunction with this trial, the parent version of the Child Health Questionnaire (CHQ) was administered [11].

Seven items from the parent CHQ that reflect similar content as that comprised in the AHUM health state classification system were used for assignment of AHUM utilities. Specifically, the concepts captured by the mapped CHQ items include eating, dressing, bathing, or going to the toilet; bodily pain or discomfort; ability to get around the neighborhood, playground, or school; satisfaction with athletic ability; general health; health rating now compared to 1 year ago; and satisfaction about looks/appearance. The selected CHQ item responses are similar to the levels of the AHUM with the exception of those for satisfaction with athletic ability and satisfaction with looks/appearance; in the CHQ, the respective responses range from ‘very satisfied’ to ‘very dissatisfied’. The responses to these items were inputted into the model to estimate utilities for each patient at each assessment time-point. The mean utilities for each treatment group were calculated for each time-point that the questionnaire was administered: baseline, 18, 36, and 53 weeks. At 53 weeks, all patients continued on weekly idursulfase therapy (placebo patients switched), and the questionnaires were again administered at 71, 89, 105, 133, and 157 weeks follow-up.

## Results

A total of 312 individuals in the UK participated in and completed the TTO interviews yielding 3,744 (12*312) observations (i.e., utilities), between 43 to 60, for each health state except for the best and worst states which had 312 observations. Based on the TTO utilities, among the 312 participants, 38 rated one of the health states in the interview as worse than the worst health state (464557), and 9 participants rated one of the health states as better than best health state (111111). One patient considered one health state, and another considered two health states, as worse than being dead, and thus these states were assigned a utility of 0.0. All participants who provided illogical ratings were included in the modeling exercise; one participant was excluded from the analysis given that, because of religious reasons, the person did not want to make choices with respect to trading time off to avoid being in hypothetical health states.

Table 2 reports the demographic characteristics of the study sample (N = 311). The participants live in a total of 47 different cities representing geographical spread across the UK. A total of 41% work full time, 42% are single, and 21% have children, and 25% have a chronic condition. A comparison of the sample statistics those reported in the UK census 2001 [20] showed that the study sample was disproportionately younger, with 24.9% versus 7.8% in the 20–24 year old age group observed in the study sample versus the census results, respectively. In addition, a higher proportion of participants in the study attained a higher education level than those reported in the census (48% vs. 27%). 

**Table 2:** Demographics of Study Population (N = 311)

**Demographics**	**N**	**(%)**
Total no. of cities represented	47	
Sex (n = 311)		
Female	144	(46.3%)
Age (n = 309)		
18-19	24	(7.8%)
20-24	77	(24.9%)
25-29	46	(14.9%)
30-44	74	(23.9%)
45-59	68	(22.0%)
60+	20	(6.4%)
Education (n = 309)		
< 5 GCSE	37	(12.0%)
5 GCSE	32	(10.4%)
2 A/AS	91	(29.4%)
Degree+	149	(48.2%)
Race/ethnicity (n = 306)		
White	248	(81.0%)
Indian	13	(4.2%)
Asian	32	(10.5%)
Other	13	(4.3%)
Relationship status (n = 310)		
Married/partner	170	(55.0%)
Single	140	(45.0%)
Have children < 18 years (n = 309)		
Yes	140	(20.7%)
No	170	(79.3%)
Occupation (n = 310)		
Full time	127	(41.0%)
Part time	28	(9.0%)
Student	102	(32.9%)
Retired	14	(4.5%)
Stay at home	10	(3.2%)
Other	29	(9.4%)
Co-morbid conditions (n = 311)		
Respiratory conditions	13	(4.2%)
GI problems	12	(3.9%)
Other chronic conditions	52	(16.7%)
No Chronic conditions	234	(75.2%)

Table 3 reports the mean rankings for each of the health states (participants were asked to rank order the health states from most to least favorable before they were asked the TTO questions) and the respective mean utilities for a sample of AHUM health states. As shown, the utilities generally become less favorable (lower) with lower rankings. The average utilities for the best health state (111111) and worse health state (454577) were 0.99 and 0.41, respectively. The utility for 111111 of 0.99 helps confirm that this state and full health largely were comparable. A utility of 0.41 for the worse health state means that the respondents would be willing to trade off 59% of their time remaining to avoid being in the worse health state. Of the 62 health states for which utilities were obtained, 12 were rated as bad as being dead (utility = 0.0) by at least one participant (3 states were considered as worse than death). All health states with a utility of 0.0 were included in the statistical model.

**Table 3:** Rankings and Time Trade Off utilities for sample of AHUM health states

**AHUM**	**Health state rankings**			**TTO utilities**				
**Health state**	**N**	**Mean**	**Median**	**Mean**	**SD**	**Min**	**Max**	**Median**
Full health	311	1.04	1	-	-	-	-	-
111111	311	2.02	2	0.99	0.06	0.10	1.00	1.00
111331	48	3.08	3	0.96	0.11	0.43	1.00	1.00
113244	60	3.67	3	0.94	0.10	0.55	1.00	0.98
131123	53	4.34	4	0.93	0.09	0.60	1.00	0.98
133322	43	4.40	4	0.91	0.12	0.50	1.00	0.98
222343	43	4.86	4	0.90	0.15	0.40	1.00	0.98
321412	43	4.91	5	0.89	0.14	0.50	1.00	0.95
222221	48	5.06	4	0.89	0.15	0.28	1.00	0.95
132213	53	5.08	5	0.92	0.09	0.70	1.00	0.95
211126	51	5.27	5	0.92	0.11	0.63	1.00	0.95
121142	53	5.32	5	0.91	0.14	0.40	1.00	0.95
312112	56	5.48	5	0.88	0.16	0.10	1.00	0.95
112435	56	5.77	5	0.90	0.15	0.10	1.00	0.95
212213	51	5.80	4	0.90	0.11	0.60	1.00	0.93
121146	51	5.84	6	0.87	0.17	0.20	1.00	0.93
211244	56	6.14	6	0.90	0.15	0.10	1.00	0.95
313221	51	6.25	5	0.86	0.14	0.50	1.00	0.90
331247	43	6.26	6	0.79	0.20	0.30	1.00	0.83
313236	48	6.31	6	0.85	0.18	0.30	1.00	0.93
131363	51	6.45	7	0.85	0.16	0.40	1.00	0.90
241232	56	6.52	6.5	0.88	0.16	0.10	1.00	0.95
113522	53	6.60	6	0.87	0.17	0.10	1.00	0.95
434131	60	6.83	6.5	0.76	0.21	0.20	1.00	0.80
253115	60	6.88	6.5	0.71	0.24	0.00	1.00	0.80
211154	56	6.95	6.5	0.88	0.15	0.10	1.00	0.93
412427	60	6.97	6.5	0.78	0.20	0.23	1.00	0.82
221242	51	7.14	7	0.85	0.16	0.30	1.00	0.93
441225	60	7.20	7	0.76	0.22	0.15	1.00	0.84
232434	56	7.23	7	0.85	0.18	0.10	1.00	0.90
342314	56	7.50	8	0.82	0.19	0.10	1.00	0.90
252326	60	7.57	8	0.73	0.23	0.00	1.00	0.80

*TTO* = Time trade off.

### Statistical model

The first iteration of the mixed effects model showed the coefficients for ‘you feel neutral about your ability to do vigorous activities’ (level 3) and ‘you feel somewhat bad about your ability to do vigorous activities’ (level 4) to be the same, and thus these 2 levels were combined for the final model, which overall was statistically significant (p < 0.0001; adjusted R2 = 0.361; RMSE = 0.194). With respect to the AHUM classification system, the dimensions clearly worsen with each subsequent level except for health perceptions, in which each level has two concepts (health compared to one year ago and current health status) that may impact preferences differently.^a^ The model showed that the coefficients differed in the expected direction across the levels of the five dimensions that have expected differences in severity except for two coefficients, the 3^rd^ level of pain – mild pain and the 3^rd^ level of self-image – feel neutral about body, which were slightly higher (lower disutility) than those for the coinciding less severe levels, respectively. Table 4 shows an example of how the model is used to compute the AHUM utility weight for health state 214524.

**Table 4:** Computing AHUM utility for health state 214524

**Dimension level**	**Health state description**	**Beta coefficient**
2	You are limited a little in eating, dressing, bathing, or using the toilet.	−0.028
1	You have no pain.	−0.0
4	You are limited a lot in walking around or going to school/work.	−0.085
5	You feel very bad about your ability to do strenuous activities (like running or participating in sports).	−0.076
2	On the whole, you feel somewhat good about your body.	−0.007
4	Your health is the same now as one year ago, and you feel like you are in good health.	−0.015

Equation for calculating AHUM utility for health state 214524.

AHUM utility = 0.976 (intercept) – 0.028 – 0.0 – 0.085 – 0.076 – 0.007 – 0.015

AHUM utility = 0.765.

### Application to a pediatric clinical trial

**Figure 1:**
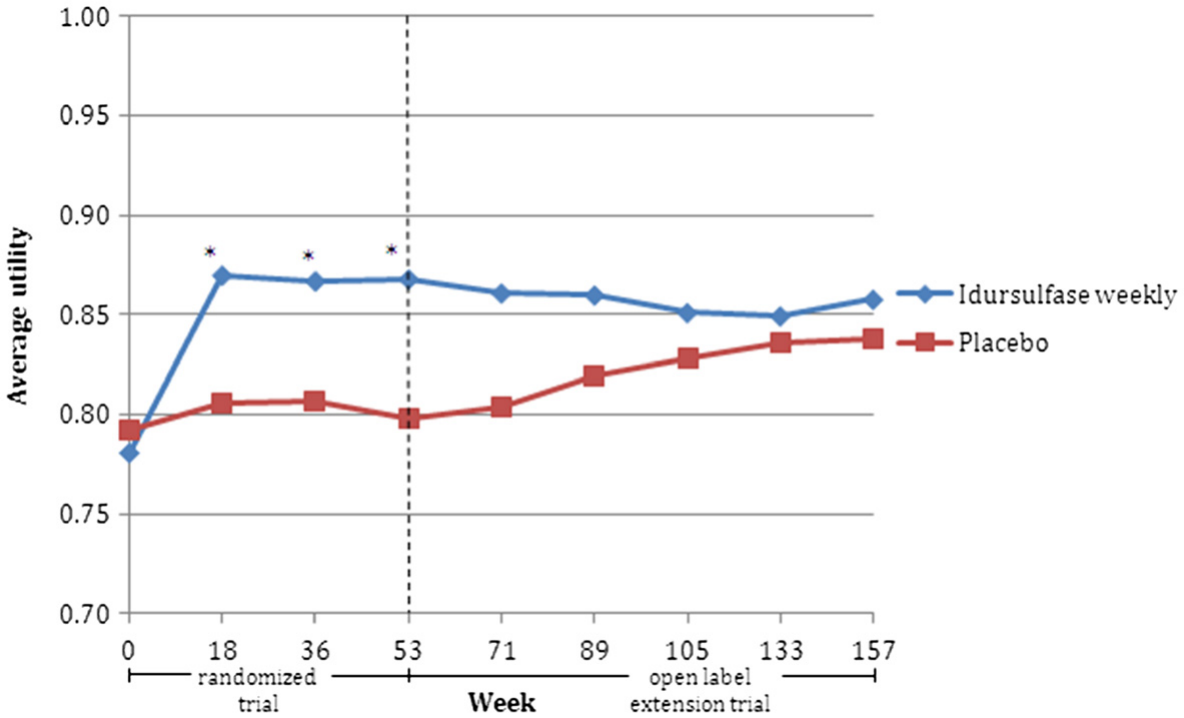
**AHUM Utilities Assigned to Pivotal & Extension Idursulfase Trial (Weeks 0 to 157): Perspective of UK Population.** *p < 0.03 for treatment group differences in mean average utility at weeks 18, 36, & 53.

The clinical trial used in this study was NCT00069641 (A phase II/III, randomized, double-blind, placebo-controlled study evaluating the safety and efficacy of weekly and every other week dosing regimens of iduronate-2-sulfatase enzyme replacement therapy in patients with MPS II) and its extension study. The random effects model was used to assign utilities to patients at each assessment time-point in the enzyme replacement therapy trial [18,19]. Specifically, the parent-completed version of the CHQ was administered during the trial, and seven items from this questionnaire that reflect similar content as that comprised in the AHUM health state classification system were used for assignment of AHUM utilities. Figure 1 shows the average utility by week from the perspective of the total population. The mean utilities in the idursulfase weekly group improve from 0.78 at baseline to 0.87 at week 53 (difference = +0.087), whereas the utilities in the placebo group improved from 0.79 to 0.80 (difference = +0.006). At baseline, the mean between the two groups were not significantly different from each other (p = 0.73). However, the group means were significantly different from each other at weeks 18, 36 and 53 (p < 0.03). In the extension phase, the mean utilities in the idursulfase weekly group remained stable over time, whereas the placebo group, which transitioned onto idursulfase therapy during this period, improved from 0.80 at week 53 to 0.84 at week 157 (difference = +0.039). It should be noted that utility weights reflect preference values and represent only one of the many types of outcomes that may be used to assess the effectiveness of treatment. 

## Discussion

The findings from this research yielded a health state classification system, the Adolescent Health Utility Measure (AHUM), that describes key impacts of having a chronic condition from the perspective of children or adolescents. The impacts largely overlap with those captured in other health state classification measures such as the SF-6D and EQ-5D, but also include self-image and health perceptions. As such, although Hunter syndrome patients were interviewed to ensure that the AHUM was comprehensive with respect to covering key areas of health from the child and adolescent perspective, the AHUM may be useful for other health conditions affecting children and adolescents. AHUM utilities may be used to quality-adjust life expectancy in future cost-effectiveness evaluation of pediatric medical interventions.

The AHUM dimensions comprise self-care, bodily pain, limitations in walking around or going to school/work, perceptions of ability to do vigorous activities, self-image, and perceptions about current health in relation to the past. As found in previous studies [6,7], these concepts have been shown to be impacted among children with chronic conditions. Based on the parameter estimates in the AHUM model, the two dimensions that appeared to be most important from the respondents’ perspectives were self care and bodily pain. For these dimensions, all severity levels, even the mildest, were statistically significant. Consistent with this finding, the EQ-5D disabilities associated with pain are highest (worse) for pain relative to the other dimensions.

Consistent with the feedback from the qualitative interviews in which children and their caregivers agree that satisfaction with current treatment/perception of making progress on treatment is important, the AHUM dimension, health perceptions, includes a description of the individual’s condition now compared to a year ago, which would be an indication of how the patient with a chronic condition is faring on treatment. Although this refers to the experience relative to one year ago, which cannot be measured absolutely, we believe that this is a critical element in fully capturing an individual’s perception of current health, particularly when one has a chronic condition and is on treatment. Previous studies have shown that knowledge that one is responding to treatment as opposed to getting worse, can significantly influence utility weights [21,22]. Poor health perceptions likely can lead to greater anxiety, which has been found to be an important concept among children with chronic conditions. For example, a study that used the General Health Questionnaire in a psychological assessment of children with mucopolysaccharidosis type II confirmed that patients and their parents had higher levels of anxiety [23].

A limitation in this study is that respondents were not able to rate health states as worse than death (reflected by a score of 0.0). Health states such as dementia and coma have been previously shown to be rated worse than death by some respondents [24]. Single index scores of the Health Utility Index (HUI) and the EQ-5D allow predicted utilities to be less than 0.0 [6,25]. The HUI2 and HUI3’s worst utilities that can be calculated are −0.03 and - 0.36, respectively [22]. In contrast, the AHUM’s worst utility weight that can be calculated is 0.419. It also should be noted that the system is based on utilities obtained from the UK population; it is possible that utility weights may vary across countries. Also, in this study the members of the general public who provided utilities were disproportionately younger compared to the general UK population as surveyed in 2001. Finally, the Health Perceptions domain does not capture all the possible combinations of health today and health last year. For future use of the AHUM, it may be useful to make assumptions *a priori* as to the assignment of utilities to this domain.

With respect to multi-attribute general health status measures, the breadth of the content typically must be limited to key concepts that broadly capture overall health-related quality of life. Similar to other multi-attribute measures, the AHUM includes key concepts that together capture overall health-related quality of life. A limitation in this study is that the AHUM domains and levels were not tested for content validity before implementation. Nevertheless, the items used in the classification system focus on concepts that are incorporated into numerous patient reported outcomes measures that have shown evidence that they are measuring the concepts they are intending to measure. The AHUM may be administered to both adults and younger subjects; however, if the AHUM will be administered to children or adolescents, then it would be useful to perform pilot testing, specifically cognitive debriefing interviews, in this population, and revise the content as necessary to ensure that the measure is understood and interpreted as expected before study implementation. Further research on the AHUM classification system would be useful, including an evaluation of the preference weights based on the perspective of an adolescent population rather than an adult population. It also would be useful to perform further research evaluating preferences for the AHUM health states in other countries where cost-effectiveness evaluations are increasingly being used to inform clinical decision making.

In conclusion, although the AHUM was developed to capture key content that may be important among older children and adolescents, this new health state classification system should apply to conditions given that the descriptive system is valid in such populations. Given that a generalizable set of health states were used, the AHUM may serve as a useful tool to derive utilities for economic evaluations of a wide range of treatments.

## Endnotes

^a^The scoring algorithm for the AHUM may be obtained from Shire AG.

## Competing of interest

K.M. Beusterien works for Oxford Outcomes Inc. which provides consulting services to Shire J.E. Yeung works for Oxford Outcomes Inc. which provides consulting services to Shire. F. Pang is an employee of Shire. J. Brazier is a paid consultant for Oxford Outcomes, which consults for Shire.

## Author’s contributions

KB directed all research activities and developed first draft of manuscript. JY managed data collection, conducted all analyses, and assisted in manuscript development. FP contributed to study design and reviewed interim drafts of the manuscript and provided comment. JB provided strategic advice on study design, analysis and interpretation of statistical model results; reviewed interim drafts of manuscript and provided comment. All authors read and approved the final manuscript.

## Funding

This research was funded by Shire Human Genetic Therapies, Inc.
